# Diagnostic potential of plasma carboxymethyllysine and carboxyethyllysine in multiple sclerosis

**DOI:** 10.1186/1742-2094-7-72

**Published:** 2010-10-29

**Authors:** Zohara Sternberg, Cassandra Hennies, Daniel Sternberg, Ping Wang, Peter Kinkel, David Hojnacki, Bianca Weinstock-Guttmann, Frederick Munschauer

**Affiliations:** 1 Department of Neurology, Baird MS center, Jacobs Neurological Institute, 100 High St. Buffalo, NY, 14203, USA; 2 Biopolymer & Proteomics Resources Roswell Park Cancer Institute Buffalo, NY 14263, USA; 3 Kinkel Neurologic Center, 5 Limestone Drive, Williamsville, NY 14221, USA

## Abstract

**Background:**

This study compared the level of advanced glycation end products (AGEs), *N*-(Carboxymethyl)lysine (CML) and *N*-(Carboxyethyl)lysine (CEL), in patients with multiple sclerosis (MS) and healthy controls (HCs), correlating these markers with clinical indicators of MS disease severity.

**Methods:**

CML and CEL plasma levels were analyzed in 99 MS patients and 43 HCs by tandem mass spectrometry (LC/MS/MS). Patients were stratified based on drug modifying therapies (DMTs) including interferon beta, glatiramer acetate and natalizumab.

**Results:**

The level of plasma CEL, but not CML, was significantly higher in DMT-naïve MS patients when compared to HCs (P < 0.001). Among MS patients, 91% had higher than mean plasma CEL observed in HCs. DMTs reduced CML and CEL plasma levels by approximately 13% and 40% respectively. CML and CEL plasma levels correlated with the rate of MS clinical relapse.

**Conclusion:**

Our results suggest that AGEs in general and CEL in particular could be useful biomarkers in MS clinical practice. Longitudinal studies are warranted to determine any causal relationship between changes in plasma level of AGEs and MS disease pathology. These studies will pave the way for use of AGE inhibitors and AGE-breaking agents as new therapeutic modalities in MS.

## Background

Advanced glycation end products (AGEs) are a heterogeneous class of compounds formed by nonenzymatic glycation and oxidation of proteins and lipids via highly reactive intermediates [1,2]. They have the potential to interact with a specific receptor (RAGE), a member of the immunoglobulin superfamily, initiating signaling pathways that amplify inflammation and oxidative stress, and thereby leading to cellular injury and death [3].

The level of AGEs increases both during physiological aging [4] and in pathophysiological settings such as diabetes mellitus (DM) [5], rheumatoid arthritis (RA) [6] and Alzheimer's disease (AD) [7]. Accumulative data suggest that AGEs may be increased in multiple sclerosis (MS), an autoimmune disease of the CNS characterized by inflammation, oxidative stress, and demyelination, which leads to axonal injury and neurodegeneration [8].

This assumption is supported by studies showing an increase in lipid peroxidation products in the CSF, plasma, and brain of MS patients [9-11]. In addition, the upregulation of the AGE receptor in oligodendrocytes, the myelin-forming cells of the CNS, is a prerequisite for the induction of oxidative damage leading to oligodendrocyte death [12].

AGEs could contribute to neurodegeneration via multiple pathways. By upregulating inflammatory cytokines and reactive oxygen species, AGEs have the potential to induce microglial activation [13]. Both oligodendrocytes death and macroglial activation are early stages of lesion formation in MS [14]. In addition, AGEs could cause neuronal cell death directly, independent of their effect on oligodendrocyte and microglial cells, since the addition of AGEs to cultured rat cortical neurons leads to a dose-dependent increase in cell death [15], which could in turn be neutralized by the addition of an AGE-specific antibody [15].

Iron deposition in the MS brain [16] could accelerate AGE formation [17], contributing to an increase in blood brain barrier permeability [18], an early and a critical event in MS pathology [19]. Since RAGE expression is positively regulated by AGEs [20], accelerated AGE production could lead to sustained RAGE expression and the amplification of inflammatory responses [21]. The involvement of AGEs in MS pathology is further supported by a study showing a relationship between the polymorphism of glyoxalase I, the gene encoding anti-glycation defense, and MS susceptibility [22].

This study was intended to determine the plasma levels of two well characterized AGEs, *N*-(Carboxymethyl)lysine (CML) and *N*-(Carboxyethyl)lysine (CEL), in MS patients and healthy control subjects, and to determine whether CML and CEL could be used as serum markers of disease activity/severity through correlation with clinical indicators of the MS disease including the Extended Disability Status Scale (EDSS), MS Severity Scale (MSSS), disease duration, and the rate of clinical relapse in the two years preceding the time of blood draw.

## Methods

### Population

Ninety-nine MS patients (71 females, 28 males, age 46.0 ± 11.5 years) were recruited from the Baird MS Center, Department of Neurology, Jacobs Neurological Institute, Buffalo, NY and from Kinkel Neurologic, Amherst, NY. MS patients were compared with 43 healthy controls (HCs) of similar age (32 females, 11 males) who were recruited from the staff of the Neurology Department. EDSS for MS patients ranged from 1 - 8.5 (mean 4.1 ± 2.2) with disease duration of 1 to 40 years. Patient population included 65 relapsing-remitting (RR), 18 secondary progressive (SP), and 9 primary progressive (PP) MS patients, along with 7 patients with clinically isolated syndrome (CIS) suggestive of MS.

Among MS patients, 44 were treated with one of the disease modifying therapies (DMTs) including interferon beta, glatiramer acetate, and natalizumab; 55 were naïve to these drugs. MS patients who received steroid treatment within one month of blood draw, those who were pregnant at the time of blood draw, and those with other autoimmune diseases were excluded from the study. In addition, patients with diabetes were excluded due to the higher AGE plasma levels observed in these patients [23,24]. Patients on statins were excluded from the study due to the statins' possible effect in lowering AGE plasma levels [25].

Among the 99 MS patients, 29 were taking one of a group of antihypertensive agents including beta-blockers, ace inhibitors and angiotensin receptor type 1 antagonist, while 25 of the 99 were on antidepressants. Other common medication included antibiotics, provigil, baclofen, albuterol, stool softeners, vitamins, and fish oil.

This study was approved by the Internal Review Board of the University at Buffalo, Buffalo, NY. Patients signed consent forms before their blood was drawn.

### Reagents

All reagents including H_2_O, 6N HCl, trichloroacetic acid (TCA), boric acid, sodium borohydride (NaBH_4)_, and nonafluoropentanoic acid (NFPA) were of analytical grade and were obtained from Sigma. CML, CEL, D_4_-CML, and D_4_-CEL were purchased from Dr. Herman Ten Brink (VU Medical Center, Metabolic Laboratory, Amsterdam, The Netherlands).

### Measurements

Plasma samples were obtained, and were frozen within 15 minutes of blood draw at -80°C until assayed. Plasma levels of CML and CEL were measured in the protein fraction by stable-isotope dilution tandem mass spectrometry described by Teerlink, *et al *[26]. Briefly, plasma samples were treated with100 mM NaBH_4 _in 200 mM borate buffer, pH 9.2 for 2 hours at room temperature. Proteins were precipitated by 200 g/L TCA. After the addition of 4.0 μM D_4_-CML and 4.0 μM D_4_-CEL, samples were hydrolyzed in 6N HCl at 110°C for 20 hours. At completion of the hydrolysis reaction, samples were blown dry under a stream of N_2 _gas at 85°C, then diluted and neutralized with 5 mM NFPA, filtered through a 0.45 μm-pore filter, and stored at -20°C. Calibration standards were prepared by mixing 1:1: 8 ratio of CML-CDL with D_4_-CML-D_4_-CEL and 5 mM NFPA respectively.

LC/MS/MS analyses were performed on an API 3000 quadropole LC/MS/MS Mass Spectrometer (AB Sciex Instruments) interfaced with an Agilent 1100 HPLC. Mass spectra were acquired and processed with Analyst 1.4.2 software (AB Sciex Instruments). LC/MS/MS conditions: samples were analyzed at 40°C by reversed phase HPLC on a Waters Symmetry C18 column, 2.1 × 150 mm, 5 μm. Mobile phase A was 5 mM NFPA in water and mobile phase B was acetonitrile. Injection volume was 20 μL with a flow rate of 220 μL/min. Compounds were eluted with a linear gradient. From 0 to 1 min mobile phase B was at 10%; from 1 min to 12 min mobile phase B was increased to 80%; after hold at 90% B for 4 min; the percentage of B was reduced to initial condition and hold for 4 min to bring the column to equilibrium.

The column effluent was introduced into a Turbo ion spray source. API3000 was operated in positive mode with the ion spray voltage at 4500 V; the decluster potential was 21V, and focusing potential was 240 V. The source temperature was 450°C. For CML and D_4_-CML, the transition of *m/z *205.1/84.0 and *m/z *209.2/88.0 were monitored; for CEL and for D_4_-CEL, the transition of *m/z *219.0/84.0 and *m/z *223.0/88.0 were monitored. The calibration range for CML and CEL was from 0.005 to 0.5 μmol/ml and was obtained by linear regression of a plot of the analyte/internal standard peak-area ratio vs. analyte concentration. The CML and CEL were expressed as ng/ml.

### Quality control tests

1. To prevent inter-assay variations, samples from both MS patients and normal controls were continually assayed in the same batch over 48 hours.

2. A pooled sample of MS patients and HCs was assayed at different time points over the course of the run to assure consistency in instrument performance.

3. Both 1:1diluted and non-diluted samples were assayed to rule out any concentration effect on the behavior of the studied compound during mass spectrometry.

4. Because of the prevalence of diabetes, and because plasma levels of AGEs have been shown to be elevated in diabetic subjects [23,24], plasma samples were randomly selected from both MS and normal groups and were assayed for HgA1c levels in order to rule out the possibility of pre-diabetic conditions affecting CML and CEL plasma levels. HgA1c plasma levels were measured in the clinical laboratories of Buffalo General Hospital, Buffalo, NY.

### Statistics

All statistical analyses were performed using SPSS 14.0 for Windows (SPSS Institute, Chicago, IL, USA). Analysis of variance (ANOVA) co-varied for age and gender was used to test the significance of differences in plasma CML and CEL between HCs, DMT-naïve MS patients and MS patients on DMTs. ANOVA also examined the differences in CML and CEL plasma levels among MS patients at different disease stages. A simple t-test assessed the differences in CML and CEL plasma levels between clinically stable MS patients and MS patients in clinical relapse, as well as differences in the percent of HgA1c between MS patients and HCs. The relationship among MS indicators of disease severity (EDSS, MSSS, disease duration and the rate of clinical relapse two years prior to blood draw), and CML and CEL plasma levels was examined using a linear regression model. P ≤ 0.05 was considered significant.

## Results

### Quality control tests

The coefficient of variation among the pooled samples was 2.0% for CML and 2.1% for CEL. The coefficient of variation for diluted vs. non diluted samples was 4.6% for CML and 5.7% for CEL. The percent HgA1c was similar between MS patients (5.25 ± 0.1%) and HCs (5.28 ± 0.1%) (p = 0.83), suggesting the absence of pre-diabetic conditions in either MS patients or HCs.

### Plasma levels of CML and CEL in MS patients and HCs

**Figure 1:**
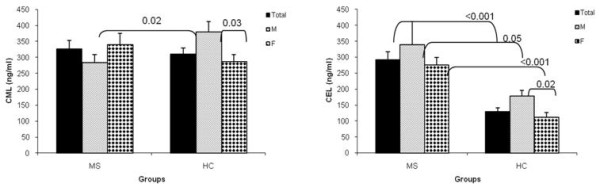
**CML and CEL plasma levels in DMT-naïve MS patients and healthy controls**: Comparison of plasma levels of CML (A) and CEL (B) in DMT-naïve MS patients and HCs, stratified by gender. The results are means ± SEM. P value ≤ 0.05 is statistically significant. Abbreviations as in Table 1 and DMT:disease modifying therapy.

Table 1 presents CML and CEL plasma levels in 43 HCs and 99 MS patients, stratified by gender and disease stage, and the associated p-values. Due to the possible effect of DMTs on CML and CEL plasma levels, all other analyses were conducted in DMT-naïve MS patients. Figure 1A compares CML plasma levels in DMT-naïve MS patients and HCs stratified by gender. CML plasma levels were not different between MS patients (325.6 ± 26.8 ng/ml) and HCs (309.5 ± 20 ng/ml) (p = 0.6). Among MS patients, 25/55 (45.4%) had a higher level of plasma CML when compared to the mean plasma level observed in HCs. Gender stratification showed a higher CML plasma level in HC males (379.7 ± 32 ng/ml) compared to HC females (285.4 ± 23 ng/ml) (p = 0.03), while plasma CML levels tended to be lower in male MS patients (283.1 ± 26.1 ng/ml) compared to females (339.7 ± 34.6 ng/ml) (p = 0.3). These gender differences led to a lower CML plasma level in male MS patients when compared to male HCs (p = 0.02).

**Table 1:** CML and CEL plasma levels in 43 HCs and 99 MS patients stratified by disease stage.

Groups	Gender	(*n*)	CML (ng/ml)	p value	CEL (ng/ml)	p value
**HC**	M+F	43	309.5 ± 20(104-683)		128.8 ± 12(17-259)	
	M	11	379.7 ± 32(215-560)		178.4 ± 16(49-258)	
	F	32	285.4 ± 23(104-683)	0.03	111.7 ± 14(17-259)	0.02
						
**MS**	M+F	99	306.4 ± 15 (117-911)		231.7 ± 15(29-781)	
	M	28	271.3 ± 17 (124-418)		237.8 ± 44(29-781)	
	F	71	319.2 ± 19 (117-911)	0.1	225.8 ± 15(34-651)	0.7
						
**CIS**	M+F	7	336.5 ± 44(182-529)		218.4 ± 79(42-502)	
	M	4	317.2 ± 45 (182-372)		171.5 ± 106(45-488)	
	F	3	362.5 ± 95(197-529)	0.6	281.0 ± 132(42-502)	0.5
						
**RRMS**	M+F	65	317.8 ± 17(140-869)		246.3 ± 20(47-781)	
	M	14	291.5 ± 21(145-418)		305.2 ± 68(47-781)	
	F	51	322.0 ± 22(140-869)	0.4	229.1 ± 17(49-651)	0.1
						
**SPMS**	M+F	18	275.4 ± 52(117-911)		195.9 ± 33(29-460)	
	M	6	197.8 ± 28(124-271)		164.1 ± 73(29-327)	
	F	12	314.6 ± 75(117-911)	0.3	206.5 ± 39(34-460)	0.6
						
**PPMS**	M+F	9	256.0 ± 20(151-362)		196.9 ± 28(53-362)	
	M	4	246.7 ± 46(151-362)		238 ± 71(115-362)	
	F	5	263.3 ± 22(217-334)	0.7	205 ± 10(176-225)	0.8
						
**RRMS (clinically stable)**	M+F	49	302.9 ± 17(140-651)		263.4 ± 25(47-781)	
	M	13	293.6 ± 24(145-418)		302.6 ± 79(47-781)	
	F	36	308.5 ± 22(140-651)	0.7	245.1 ± 21(49-651)	0.3
						
**RRMS (in clinical relapse)**	M+F	16	362.5 ± 42(158-869)		196.6 ± 22(74-442)	
	M	1	338.00		204.4	
	F	15	364.4 ± 45(158-869)		196.1 ± 24(75-442)	

CML and CEL plasma levels in 43 HCs and 99 MS patients stratified by both disease stage and gender. The results are mean ± SEM and associated ranges. P values are related to gender differences in CML and CEL plasma levels. P value ≤ 0.05 is statistically significant.

Figure 1B compares CEL plasma levels between DMT-naïve MS patients and HCs stratified by gender. CEL plasma levels were 122% higher in MS patients (291.7 ± 25 ng/ml) compared to that in HCs (128.8 ± 12 ng/ml) (p < 0.001). CEL plasma levels were significantly higher in HC males (178.4 ± 16 ng/ml) compared to HC females (111.7 ± 14 ng/ml) (p = 0.02), and tended to be lower in male MS patients (338.8 ± 72 ng/ml) compared to females (276.1 ± 23 ng/ml) (p = 0.3). Approximately 91% (50/55) of MS patients had plasma CEL levels above the mean observed in HCs.

### Effects of DMTs on CML and CEL plasma levels

**Figure 2:**
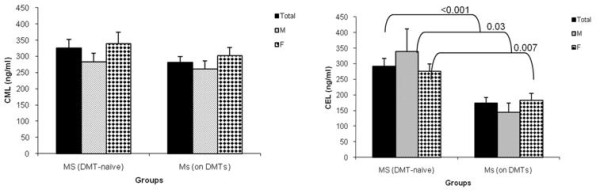
**CML and CEL plasma levels in DMT-naïve MS patients and MS patients treated with DMTs**. Comparison of Plasma levels of CML (A) and CEL (B) in DMT-naïve MS patients and patients on DMTs, stratified by gender. The results are means ± SEM. P value ≤ 0.05 is statistically significant. Abbreviations as in Table 1, and DMT:disease modifying therapy.

Figure 2A and Figure 2B respectively compares CML and CEL plasma levels between DMT-naïve MS patients (n = 55) and those patients on DMTs (n = 44). Patients' stratification based on their DMT regimen showed 13.7% lower plasma CML in MS patients treated with DMTs (280.9 ± 18 ng/ml) compared to patients naïve to DMTs (325.6 ± 26.8 ng/ml) (p = 0.2) (Figure 2A). The DMT regimen led to a reduction of 7% in plasma CML levels in males (from 283.1 ± 26.1 ng/ml to 261.9 ± 24.8 ng/ml, p = 0.7) and 11% in females (from 339.7 ± 34.6 ng/ml to 301.8 ± 26 ng/ml, p = 0.4).

Patients treated with DMTs had 40.5% lower plasma CEL (173.4 ± 18 ng/ml) compared to DMT-naïve patients (291.7 ± 25 ng/ml, p < 0.001) (Figure 2B). However, MS patients on DMTs still presented with a significantly higher CEL plasma level compared to HCs (p = 0.04). The percent reduction in CEL plasma levels due to DMTs, was higher in males (57.4%, from 338.8 ± 72.1 ng/ml to 144.3 ± 29.6 ng/ml, p = 0.03) compared to females (33.7%, from 276.1 ± 23.7 ng/ml to 183.0 ± 21.8 ng/ml, p = 0.007).

In addition, MS patients who were on antihypertensive drugs had lower plasma CML (279.3 ± 24.1 ng/ml) compared to patients who were not on this regimen (324.8 ± 26.0 ng/ml) (p = 0.4). Similarly, MS patients who were on antihypertensive drugs had lower plasma CEL levels (229.1 ± 57.0 ng/ml compared to patients who were not on this regimen (287.7 ± 25.1 ng/ml) (p = 0.3).

Antidepressants were an additional common group of drugs that MS patients were treated with at the time of blood sampling. However, we did not observed any effect of antidepressants on plasma levels of CML or CEL (results are not shown).

### CML and CEL plasma levels in MS patients at different disease stages

**Figure 3:**
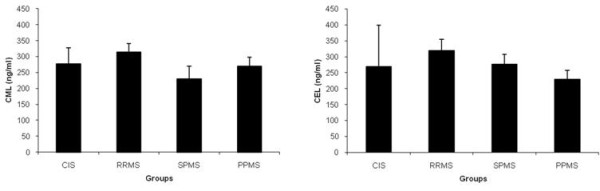
**Plasma CML and CEL levels in DMT-naïve MS patients at various disease stages**. Plasma CML (A) and CEL (B) at various disease stages, in DMT-naïve, clinically stable MS patients. Results are means ± SEM for 4 CIS patients, 28 RRMS patients, 8 SPMS patients and 7 PPMS patients. Abbreviations as in Table 1.

Due to the lowering effects of DMTs, CML and CEL plasma levels were compared in DMT-naïve, clinically stable MS patients, at various disease stages. The level of plasma CML was not different between RRMS patients (n = 28) (314.3 ± 25.6 ng/ml) compared to SPMS patients (n = 8) (229.7 ± 39.4 ng/ml), PPMS patients (n = 7) (270.1 ± 27 ng/ml) and CIS patients (n = 4) (276.7 ± 50 ng/ml) (ps >0.05) (Figure 3A). Similarly, the level of plasma CEL was not different between RRMS patients (319.0 ± 35 ng/ml) compared to SPMS patients (276.2 ± 30.9 ng/ml) and CIS patients (269.4 ± 130 ng/ml) (Figure 3B), but CEL plasma level tended to be lower in PPMS patients (229.8 ± 28 ng/ml) compared to RRMS patients (p = 0.08)

### CML and CEL plasma levels in clinically stable MS patients and MS patients in clinical relapse

Since there was only one male MS patient who had clinical relapse at the time of blood draw, we compared clinically stable female RRMS patients (n = 25) with female RRMS patients in clinical relapse (n = 7). CML plasma levels tended to be higher in DMT-naïve MS patients who were in relapse (429.8 ± 88.6 ng/ml) compared to similar group of patients who were clinically stable (306.0 ± 29.8 ng/ml) (p = 0.09). However, CEL plasma levels were not different between patients in clinical relapse (274.0 ± 59.1 ng/ml) compared to clinically stable MS patients (260.8 ± 21.4 ng/ml) (p = 0.8).

### Correlation between plasma CML and CEL and indicators of MS disease severity

There was no correlation between age and plasma CML or CEL levels in either MS patients or normal subjects. When measured in DMT-naïve patients, CEL plasma levels (R = 0.25, Rsqr = 0.06, Adj Rsqr = 0.05, p = 0.01) correlated with the rate of clinical relapse, during the two years before the time of blood sampling, more significantly than CML plasma levels (R = 0.23, Rsqr = 0.05, Adj Rsqr = 0.04, p = 0.04). No significant correlation between CML and CEL plasma levels, and EDSS and MSS was observed.

## Discussion

This study examined plasma levels of CML and CEL, two well-characterized AGEs, in MS patients and HC subjects. After ruling out the possibility of any pre-diabetic conditions in the study population, we found significantly higher plasma levels of CEL in MS patients compared to HCs. CML plasma levels were not different in MS patients compared to HCs, but similar to CEL, CML plasma levels correlated with the rate of clinical relapse, measured the two years before blood sampling, suggesting that both CML and CEL may be involved in MS pathology. This conclusion is further supported by immunohistochemical studies in the authors' laboratory showing increased immunostaining of CML and its receptor RAGE in postmortem hipocampi slides derived from MS patients when compared to those from HCs [27].

Since AGEs promote inflammation and oxidative stress [28], higher levels of AGEs in MS patients suggest higher inflammatory state, hence predisposing these patients to a higher rate of yearly clinical relapse.

The increase in AGE plasma levels in MS patients could be due to either leakage resulting from blood-brain barrier dysfunction [29] or it can stem from the activation of patients' peripheral immune cells due to chronic ongoing inflammation and oxidative stress [30].

CML and CEL have inflammatory effects via signaling through the RAGE receptor [3]. Both agents are formed through Maillard reaction, which combines reduced sugars with the amino-acid lysine to form glycated adducts called Amadori products. These byproducts can then undergo further intramolecular rearrangement to produce the two AGEs. However, in addition to glycoxidation, lipid oxidation can also contribute to the formation of CML and CEL [31].

CML can be formed via multiple pathways including the reactive intermediate glyoxal, but the primary pathway for CEL formation is a nonenzymatic reaction between the reactive intermediate methylglyoxal, formed by protein glycoxidation, or enzymatic glycolysis with lysine residues [32,33]. Both glyoxal [34] and methylglyoxal [35] have been shown to be neurotoxic, but they have been shown to induce cellular death via different signaling pathways [36], suggesting that CML and CEL may be involved in different aspects of MS pathology.

The other significant result of this study was the lowering effect of DMTs on plasma AGEs. The level of plasma CEL was lower in MS patients on DMTs and in HCs compared to DMT-naïve patients. In addition, CEL plasma levels were more significantly affected by DMTs than CML plasma levels, and more significantly in male patients than in females. It is known that men have a more severe MS disease with usually a higher rate of disease progression. Future larger studies evaluating the AGES plasma levels in relation to gender may shed a light in understanding of disease heterogeneity leading to a more specific individualized therapeutic interventions.

Despite the correlation between the plasma level of AGEs and the rate of clinical relapse, and the notion that clinical relapse represents a heightened inflammatory status [37], one could not observe a significantly higher plasma level of AGEs in patients during relapse when compared to clinically stable MS patients.

Although our results regarding the effect of relapse on plasma CML and CEL levels are not conclusive, due to a small sample size of relapsing patients who were naïve to DMTs (n = 7), one should note that AGE accumulation in tissues is attributed to chronic ongoing inflammation. Assuming that in average MS patients experience 1-2 clinical relapses per year, with each lasting no more than two weeks, relapse time does not last long enough for accumulation of AGEs in the blood. The measurement of plasma AGEs in the same patient both during clinical relapse and during remission may give a better indication for the usefulness of AGEs as predictive markers of clinical relapse.

Our results with MS patients are consistent with earlier in vitro [38] and animal [39] studies showing that antihypertensive drugs reduce the level of AGEs. However, this is the first study reporting the effect of DMTs on lowering plasma AGEs in MS patients. These results suggest that DMTs may exert therapeutic effects in part through modulation of AGEs. The higher level of AGEs in MS can affect the expression of the RAGE receptor, promoting downstream inflammatory and neurodegenerative processes [40]. Interestingly, these agents tend to reduce CEL levels mainly in males, suggesting that gender differences may be involved in the therapeutic effects of DMTs [41,42].

Since AGEs are markers of inflammation and oxidative stress, they are not specific to MS, but have been shown to be upregulated in other neurodegenerative diseases such as Alzheimer's disease [43]. Furthermore, AGEs have been shown to be involved in a number of demyelinating diseases. AGE-modified peripheral nerve myelin is susceptible to phagocytosis by macrophages contributing to demyelination and peripheral neuropathy in diabetic patients [44]. Furthermore, immunohistochemical studies observed the upregulation of AGE CML in the neurons and in microglia of spinal cord of patients suffering from amyotrophic lateral sclerosis [45].

Although this study did not measure the level of total lysine-containing proteins, the results of a two-dimensional electrophoretic procedure did not observe differences in plasma proteins between MS patients and normal control subjects [46].

## Conclusion

Our results suggest that plasma AGEs may provide a simple and novel diagnostic tool in MS. This is especially true with CEL levels, where 91% of patients were identified as having higher than normal levels of AGEs. Future studies with larger cohort will be needed to assess AGEs usefulness as serum biomarkers in MS, and whether these markers could supplement current monitoring strategies in clinical trials.

Assessment of AGE involvement in MS pathology will pave the way for the introduction of AGE inhibitors or AGE breaking agents in MS clinical practice. Phase II clinical trial of the AGE breaker ALT-711 conducted in 93 individuals over the age of 50 with evidence of vascular stiffening showed reduction in arterial pulse pressure and increase in large artery compliance [47]. AGE inhibitors have been also suggested as promising neuroprotective strategies [48].

## Abbreviations

CML: carboxymethyllysine; CEL: carboxyethyllysine; HC: healthy control; MS: multiple sclerosis; CIS: clinically isolated syndrome; RRMS: relapsing remitting multiple sclerosis; SPMS: secondary progressive multiple sclerosis; PPMS: primary progressive multiple sclerosis; M: male; F: female; n: number of subjects.

## Competing interests

The authors declare that they have no competing interests.

## Authors' contributions

ZS: design, data analysis and interpretation, manuscript drafting; CH, DS: data acquisition; PW: Protocol optimization; PK, DH, BWG: patients' recruitment, FM: intellectual input. All of the authors have read and have approved the final version of this manuscript.
